# Effects of Arbuscular Mycorrhizal Fungi on Alleviating Cadmium Stress in *Medicago truncatula* Gaertn

**DOI:** 10.3390/plants12030547

**Published:** 2023-01-25

**Authors:** Wanting Li, Ke Chen, Qiong Li, Yunlai Tang, Yuying Jiang, Yu Su

**Affiliations:** 1 School of Life Science and Engineering, Southwest University of Science and Technology, Mianyang 621010, China; 2 Sichuan Academy of Forestry, Chengdu 610036, China

**Keywords:** cadmium stress, arbuscular mycorrhizal funji (AMF), metabolic group

## Abstract

Heavy metal contamination is a global problem for ecosystems and human health. Remediation of contaminated soils has received much attention in the last decade. Aided mitigation of heavy metal phytotoxicity by arbuscular mycorrhizal fungi (AMF) is a cost-effective and environmentally friendly strategy. This study was carried out to investigate the mitigation effect of AMF inoculation on heavy metal toxicity in *Medicago truncatula* under soil cadmium stress. Therefore, a pot experiment was designed to evaluate the growth, chlorophyll fluorescence, Cd uptake and distribution, malondialdehyde (MDA) content, root soil physicochemical properties, and metabolite profile analysis of *M. truncatula* with/without AMF inoculation in Cd (20 mg/Kg)-contaminated soil. The results showed that inoculating AMF under Cd stress might enhance photosynthetic efficiency, increase plant biomass, decrease Cd and MDA content, and improve soil physicochemical properties in *M. truncatula*. Non-targeted metabolite analysis revealed that inoculation with AMF under Cd stress significantly upregulated the production of various amino acids in inter-root metabolism and increase organic acid and phytohormone synthesis. This study provides information on the physiological responses of mycorrhizal plants to heavy metal stress, which could help provide deeper insight into the mechanisms of heavy metal remediation by AMF.

## 1. Introduction

Cadmium (Cd) is a widespread heavy metal pollutant that is highly toxic to living organisms. Pollution with Cd is a primary environmental concern worldwide because of its diffusion, persistence, and harmful effects [1]. Geogenic (natural) and anthropogenic activities are sources responsible for soil contamination by heavy metals. Heavy metals in the environment have various origins: they can arise from biological processes such as rock weathering, volcanic eruptions, forest fires, and soil-forming processes [2]. However, cadmium-contaminated soil often results from pollution, chiefly generated by human factors. Heavy metal pollution has been a severe problem since the last century. Several developed countries have suffered from severe soil contamination caused by chemical waste releases. This contamination has spread worldwide, with a significant concentration in Europe [3]. Cadmium is the most common heavy metal pollutant in agroecosystems. Its pollution sources include atmospheric deposition, pesticides and plastic films, sewage irrigation in agricultural fields, sludge fertilization, uncontrolled accumulation of heavy-metal-containing waste, heavy metal mines, and acidic wastewater pollution [4,5]. Among those sources, sewage irrigation, chemical fertilizers, and pesticides are the leading causes of cadmium pollution in agricultural soil [6]. The accumulation of the heavy metal Cd threatens environmental quality, food safety, and public health when it is far beyond the cleaning capabilities of the soil ecosystem itself [7,8]. The root part of the plant is in direct contact with the Cd-contaminated soil, thus the most significant damage is caused to the plant’s roots, which exhibit increased root color, local tissue enlargement, or even decay, under high stress concentrations [9,10]. Heavy metal ions form a competitive relationship with other mineral nutrients, leading to a decrease in various nutrients transported to the plant’s above-ground parts. This results in metabolic disturbances in plant physiology and biochemistry, wilting leaves and fruits showing dry blight [11,12,13]. People consume food crops contaminated by heavy metal cadmium throughout the food chain. Once the cadmium enters the human body, it forms cadmium sulfur protein, which enters various body parts through blood circulation. It is not metabolized well in the human body and so it accumulates, mainly in the kidneys and liver.Very little cadmium is excreted in the feces and urine. When the heavy metal cadmium accumulates to a certain amount in the human body, bones loosen, the liver and kidney organs fail, and cancer can be induced, leading to human death [14,15].

Microbial flora (fungi, bacteria, and algae) can help eliminate organic and metal contaminants [16]. The central potential benefits of microbial remediation of metals are low operating costs, high capacity, and metal recovery potential [17]. Certain fungi are plant growth promoters and biocontrol agents because they adapt to soils with high contaminants [18,19,20]. Arbuscular mycorrhizal fungi are ubiquitous in various types of ecosystem (including multiple types of polluted area, extreme environments, acid-base extremes, and salt zones). They can form symbioses with more than 80% of terrestrial plants [21]. They are widely used in agriculture, woodland, grassland, and other ecosystems because they mainly promote the uptake of nutrients by plants and can live in mutually beneficial symbiosis with the host. The rapidly growing hyphae promote symbiosis with the host plants, and these hyphae can grow under harsh environmental conditions of metal toxicity and stress. During symbiosis, AMF can induce changes in host plants in response to this toxic environment. AMF induces tolerance in plants indirectly by alleviating oxidative stress produced by heavy metals by altering the root morphology, resulting in an increased in above-ground biomass through improved water and mineral nutrient absorption [22]. Rice contaminated with metals, especially arsenic (As) and cadmium (Cd), poses serious health risks. Studies have shown that the AMF cluster can reduce the As fraction and Cd concentration in rice [23]. Cui found that AMF promoted the growth of *Suaeda salsa* and increased the accumulation of Na, Cd, and other mineral elements but decreased the above-ground Na, Cd, and malondialdehyde (MDA) contents under Cd stress. They also analyzed various regulatory pathways *Suaeda salsa* after application of AMF in [24]. AMF inoculation in soybean promoted soybean growth and seed yield by increasing phosphorus uptake and accumulation in plant tissues. Inoculation with AMF reduced metal toxicity in soybean grown in contaminated soil by retaining heavy metals in the roots, resulting in lower Cu, Pb, and Zn transport in the plant’s above-ground portion, increasing whole-plant productivity [25]. Under Ni stress conditions, AMF significantly positively affected *Lolium perenne* growth, water content, and photosynthetic pigment content, probably through an avoidance strategy leading to reductions in Ni accumulation in plant tissues and translocation of Ni to shoots [26]. In Pb-contaminated *Bidens parviflora*, inoculation with AMF significantly increased plant root Pb accumulation and growth by enhancing antioxidant defense and photosynthetic efficiency under Pb stress conditions [27]. Cui demonstrated that AMF could alleviate Cd toxicity in soybean, and AMF promoted the partitioning of mineral elements in the stems and roots of soybean [28]. Chang showed an improvement in biomass and nutritional status of plants after inoculation with AMF under Cd and lanthanum stress (N, P, and K uptake increased between 20.1% and 76.8%) and the alleviation of heavy metal toxicity was associated with reduced uptake of the heavy metals by plant organs [29]. AMF species are rich, diverse, and numerous. Single and composite mycorrhizal fungi can be widely present in various heavy-metal-contaminated soils [30]. Therefore, using AMF to remediate heavy-metal-contaminated soil has become a hot topic of interest and research for ecologists.

*Medicago truncatula*, an annual plant of the legume genus *Medicago*, has been considered a model plant for studies because of its small genome, short growth period, self-pollination, nitrogen fixation via root nodules, and high efficiency of genetic transformation. In addition, it is an excellent high-yielding forage grass. Therefore, *Medicago truncatula* as the test plant, the heavy metal Cd, and AMF were selected as the study subjects. This study focused on the effects of AMF addition on the growth, photosynthetic properties, Cd uptake, and soil physicochemical properties of *Medicago truncatula* under Cd-contaminated soil conditions. Non-targeted metabolomics was used to analyze the metabolic status of plant roots after adding AMF under Cd stress.

## 2. Results

### 2.1. Effect of Inoculation with AMF on the Growth of M. truncatula

The mycorrhizal colonization rate by *G.mosseae* of AM and Cd-AM treatment was detected as 68.51% and 53.38%, respectively. While the control (CK) and Cd treatment was detected no signs of colonization. Under normal physiological conditions, *M. truncatula* stem dry weight, stem water content, and root length were significantly increased 1.81-fold, 1.04-fold, and 1.57-fold, respectively, after AMF addition (Table 1). The water content of *M. truncatula* stems was increased 1.11-fold by inoculation with AMF under Cd^2+^ stress (Table 1). Inoculation with AMF had little effect on the roots of *M. truncatula* but the stem biomass was affected by AMF. Overall, these results indicated that AMF could regulate plant growth.

### 2.2. Effect of Inoculation with AMF on M. truncatula Chlorophyll Fluorescence

**Figure 1:**
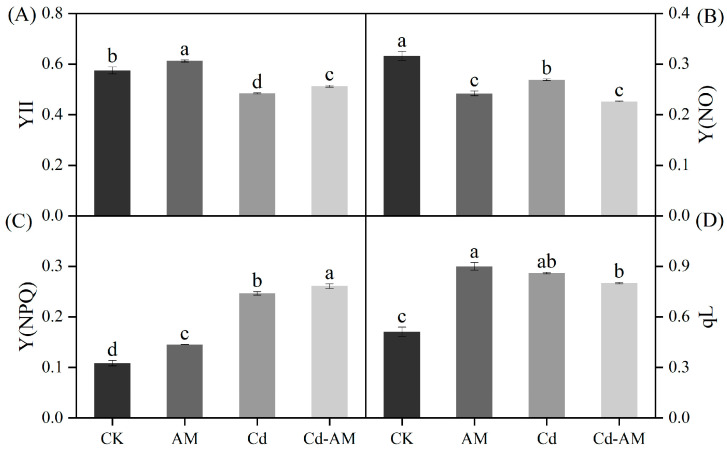
Response of *M. truncatula* leaf fluorescence parameters Y(II) (**A**), Y(NO) (**B**), Y(NPQ) (**C**), and qL (**D**) under different treatments. Values are expressed as means ± SE (*n* = 3). Values followed by the same letter in the same column are not significantly different (*p* < 0.05).

**Figure 2:**
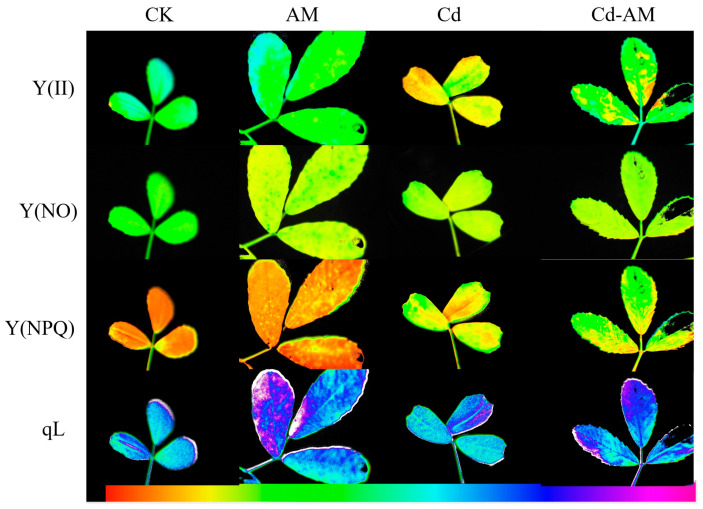
Chlorophyll fluorescence images of (II), Y(NO), Y(NPQ), and qL at steady-state with actinic illumination of 200 μmol·photons·m^−2^·s^−1^ measured at the end of the experiment in leaves of different treatments. The false color code depicted at the bottom of each image ranges from 0.000 (black) to 1.000 (pink).

Figure 1 and Figure 2 show the chlorophyll fluorescence parameters and images of leaves under CK, AM, Cd, and Cd-AM treatments. Under normal physiological conditions, inoculation with AMF significantly increased Y(II), Y(NPQ), and qL 1.06-fold,1.34-fold, and 1.76-fold, respectively. However, it significantly reduced Y(NO) by 22.8% (*p* < 0.05). Under cadmium stress, Y (II) decreased significantly. However, inoculation with AMF under Cd^2+^ stress significantly increased Y(II) and Y(NPQ) but significantly reduced Y(NO) (*p* < 0.05). This indicates that inoculation with AMF positively regulated photosynthesis in *M. truncatula* and alleviated photosynthetic organ damage under Cd^2+^ stress.

### 2.3. Effect of AMF Inoculation on the Cd^2+^ Distribution and Content of M. truncatula

The content of Cd^2+^ in different parts of *M. truncatula* is shown in Table 2. Under Cd^2+^-only treatment, the content in *M. truncatula* stem was 0.0321 mg/g, and was 0.1396 mg/g in the root. After inoculation with AMF, the content of the *M. truncatula* stem was 0.0293 mg/g, and the root was 0.0683 mg/g, significantly lower than the Cd group (*p* < 0.05). The results indicated that, in *M. truncatula,* Cd^2+^ was mainly stored in the roots, and only a tiny amount was transferred to the stems. In addition, the inoculation with AMF reduced the uptake of Cd^2+^ by *M. truncatula*, thus alleviating the toxicity and mitigating the physiological and photosynthetic damage to the plants.

### 2.4. Effect of AMF Inoculation on M. truncatula MDA Content and Antioxidant Enzyme Activity

**Figure 3:**
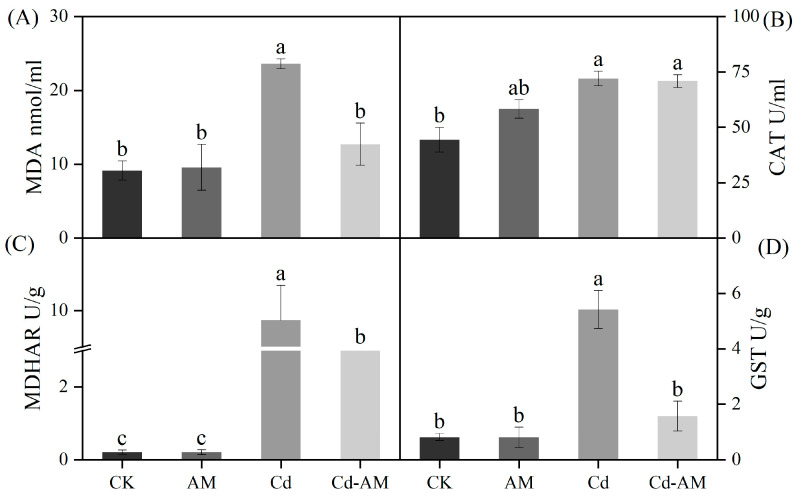
*M. truncatula* leaf malondialdehyde (MDA) (**A**), catalase (CAT) (**B**), monodehydroascorbate reductase (MDHAR) (**C**), and glutathione sulfhydryltransferase (GST) (**D**) activities under different treatments. Values are expressed as means ± SE (*n* = 3). Values followed by the same letter in the same column are not significantly different (*p* < 0.05).

Under normal physiological conditions, inoculation with AMF did not affect plants’ MDA and antioxidant activity, but inoculation with AMF under Cd^2+^ stress did affect plants. Compared with CK, the MDA content of *M. truncatula* was significantly increased, 2.5-fold under Cd^2+^ stress. Compared with the Cd group, the MDA content was significantly decreased by 53% after AMF inoculation, indicating that AMF inoculation could significantly alleviate the oxidative damage of cell membrane caused by Cd^2+^ stress (Figure 3A). Inoculation with AMF under normal physiological conditions had no significant effect on the antioxidant enzyme system of the plants. Compared with CK, catalase (CAT), monodehydroascorbate reductase (MDHAR), and glutathione sulfhydryltransferase (GST) activity increased significantly in the Cd group, indicating that the plant’s defense mechanisms were still functioning under heavy metal stress (Figure 3B–D). However, after inoculation with AMF, both MDHAR and GST activities were significantly decreased, probably because AMF reduced the toxicity of Cd^2+^ to plants (Figure 3C,D).

### 2.5. Effect of AMF Inoculation on Inter-Root Soil Properties

**Figure 4:**
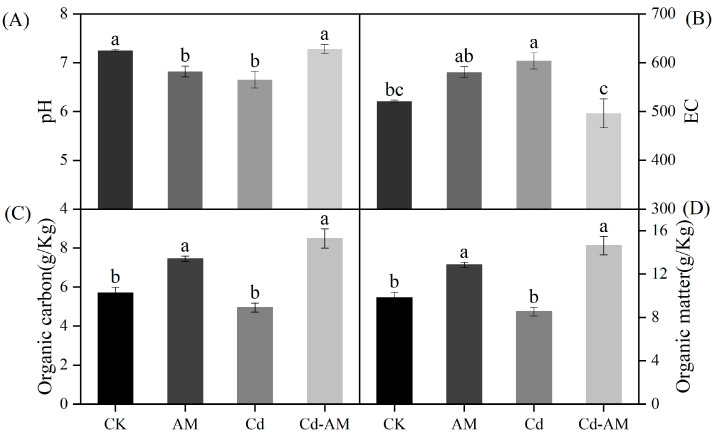
Inter-root soil physicochemical properties under different treatments. Soil potential of hydrogen (pH) (**A**), electrical conductivity (EC) (**B**), organic carbon (**C**), and organic matter content (**D**). Values are expressed as means ± SE (*n* = 3). Values followed by the same letter in the same column are not significantly different (*p* < 0.05).

Inoculation of AMF under Cd^2+^ stress showed no significant difference in soil pH and EC values compared to CK (Figure 4A,B).AMF inoculation under normal physiological conditions and Cd^2+^ stress significantly increased the soil’s organic carbon and organic matter content (Figure 4C,D). This indicates that the addition of AMF can effectively improve the essential nutrient content of the soil and have a buffering effect on the physicochemical properties of the soil under Cd^2+^ stress.

### 2.6. Effect of AMF Inoculation on the Inter-Root Metabolites of M. truncatula

To better understand the pattern of inter-root metabolite changes in *M. truncatula* under different treatments, the inter-root metabolites in the samples were identified based on GCMS non-targeted metabolomics techniques. A total of 334 metabolites were detected, including 55 carbohydrates and carbohydrate conjugates, 51 amino acids, peptides, and analogs, and 31 fatty acids and conjugates.

#### 2.6.1. Multivariate Statistical Analysis of Metabolites

**Figure 5:**
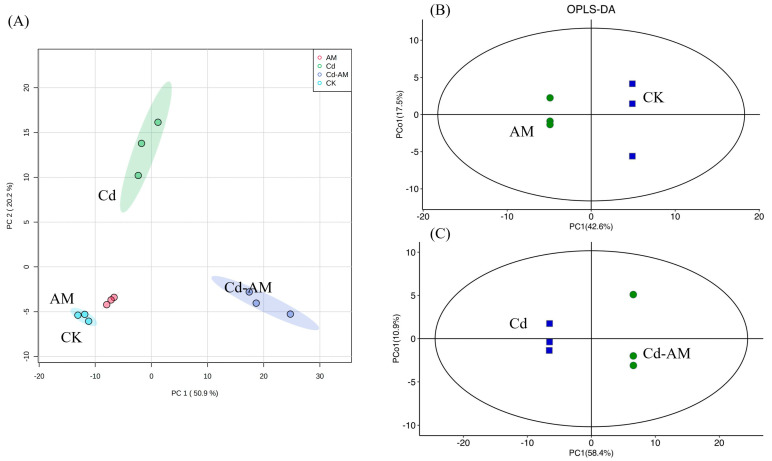
Effect of inoculation with AMF on inter-root metabolites. Principal component analysis (PCA) of the different treatments (**A**). Orthogonal partial least squares discriminant analysis (OPLS-DA) under different treatments: AM vs. CK (**B**), Cd-AM vs. Cd (**C**). The metabolomic dataset was produced through GCMS and then used for the multivariate OPLS-DA modeling (R^2^X = 0.747 Q^2^ = 0.996 (**B**) and R^2^X = 0.804 Q^2^ = 0.994 (**C**)).

PCA analysis characterizes metabolomics under multidimensional data through several principal components, so that differences between groups can be observed through PCA plots. In the present study, the separation of the different treatments was evident in the PCA results, which indicated that the inter-root metabolites in the samples changed significantly after the different treatments, appearing to be in agreement with the physiological indicators. The first principal component (PC1) could explain 50.9% of the features in the original dataset. The second principal component (PC2) could explain 20.2% of the features in the original dataset (Figure 5A).The third principal component (PC3) could explain 7.7% of the features in the original dataset. The fourth principal component (PC4) could explain 4.5% of the features in the original dataset. The fifth principal component (PC5) could explain 4.4% of the features in the original dataset. The sixth principal component (PC6) could explain 3.5% of the features in the original dataset.OPLS-DA analysis is a multivariate statistical analysis method with supervised pattern recognition, which can effectively remove the effects irrelevant to the study and thus screen for differential metabolites. The scores were plotted using OPLS-DA analysis of AM vs. CK and Cd-am vs. Cd pairs, in which R^2^X and R^2^Y represent the explanatory rates of the constructed model for X and Y matrices, respectively, and Q^2^ indicates the model’s predictive power, with all comparison groups having a Q^2^ higher than 0.5, indicating that the constructed model is appropriate. The OPLS-DA score plots indicate that a significant separation between the different comparison groups was achieved (Figure 5B). Overall, different treatments significantly affected the inter-root metabolites of *M. truncatula*.

#### 2.6.2. Differential Metabolite and Enrichment Pathway Analysis

**Figure 6:**
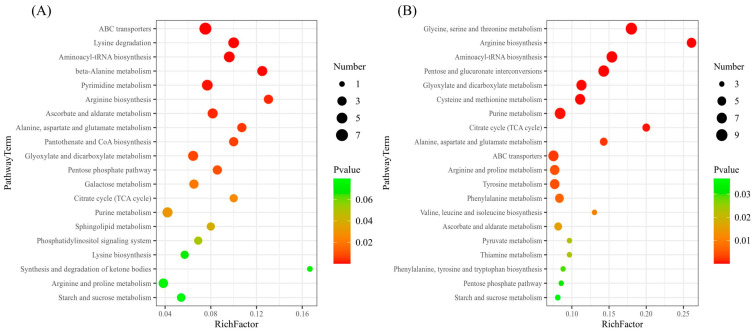
Enrichment pathway of different metabolites under different treatments. AM vs. CK (**A**), Cd-AM vs. Cd (**B**).

Furthermore, we used the VIP value and P value to screen for differential metabolites. Metabolites that met both VIP value > 1 and *p* value < 0.05 were considered differential metabolites. The results showed that AM vs. CK screened 80 differential metabolites, of which 61 were upregulated and 19 were downregulated. Cd-AM vs. Cd screened 119 differential metabolites, of which 88 were upregulated and 31 were downregulated. All the differential metabolites in the different comparison groups were matched to the KEGG database to obtain information on the pathways in which the metabolites were involved. The enrichment analysis was performed on the annotated results to obtain the pathways with high enrichment of the differential metabolites. The differential metabolites of AM vs. CK were mainly annotated and enriched in the ABC transporters pathway and the lysine degradation pathway. The differential metabolites of Cd-AM vs. Cd were mainly annotated and enriched in the glycine, serine, and threonine metabolism pathways and the arginine biosynthesis pathway (Figure 6).

#### 2.6.3. Visual Network Analysis of Inter-Root Amino Acid Metabolism

**Figure 7:**
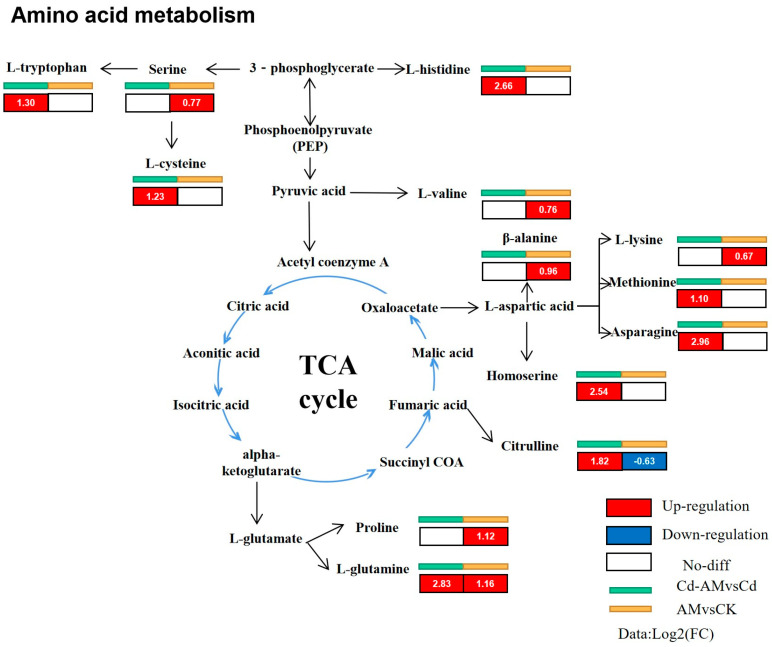
Visual analysis of rhizosphere metabolites after inoculation with AMF.

The response mechanisms of the plant basal metabolic network induced by the different treatments are shown in Figure 7. Inoculation with AMF had a significant effect on amino acid metabolism. Inoculation with AMF under normal physiological conditions significantly upregulated proline (1.12-fold), L-glutamine (1.16-fold), beta-alanine (0.96-fold), serine (0.77-fold), L-valine (0.76-fold), and L-lysine (0.67-fold). Inoculation of AMF under Cd^2+^ stress affected the metabolism of various amino acids, significantly upregulating L-asparagine (2.96-fold), L-glutamine (2.83-fold), L-histidine (2.66-fold), homoserine (2.54-fold), citrulline (1.82-fold), L-tryptophan (1.30-fold), L-cysteine (1.23-fold), and methionine (1.10-fold).

## 3. Discussion

Ecological pollution by the heavy metal cadmium has attracted extensive attention worldwide [31,32,33]. Studies have shown that under cadmium stress conditions in the soil, plants are subjected to the toxic effects of heavy metals, leading to adverse effects on growth, photosynthetic activity, and antioxidant enzyme activity [34,35]. There are more studies on cadmium remediation, and this study remediation of cadmium-contaminated soil was conducted from the perspective of the plant and microorganisms. AMF is a fungus that can be inoculated into plants to promote plant growth and alleviate plant damage under abiotic stress by increasing plant biomass, photosynthetic activity, and altering related metabolites [22,36,37]. The parameter of plant growth was used as one of the essential criteria to assess plant tolerance to Cd stress. In this study, inoculation of AMF increased the water content of *M. truncatula* stems under cadmium stress, indicating that AMF could alleviate the damage to plants under heavy metal stress [38,39]. Photosynthetic characteristics are essential when studying changes in plant physiological parameters [40,41]. Y(II) indicates the quantum yield of photosynthetic system II (PSII) photochemistry. Y(NO) is the quantum yield of non-regulatory energy dissipation in PSII and is used as a marker to indicate plant photodamage. Y(NPQ) represents the quantum yield of non-photochemical quenching in PSII and is often used as an essential indicator of plant photoprotection. qL is the share of PSII antenna pigments absorbing light energy for photochemical electron transfer based on the lake model, indicating the degree of openness of the PSII reaction centers. In the present study, inoculation with AMF under normal physiological conditions significantly increased the photosynthetic activity of plants compared with the CK group, probably by increasing the activity of photosynthetic organs [37,42]. Y(II) was significantly decreased by cadmium stress, suggesting that heavy metal stress reduced the amount of light absorbed by the reaction center [43,44]. However, inoculation with AMF under Cd stress significantly increased the actual quantum yield Y(II) and the heat dissipation level Y(NPQ) and, it significantly decreased the plant’s photodamage level Y(NO). It is suggested that inoculation with AMF may alleviate the toxicity of Cd to *M. truncatula* by increasing heat dissipation [45,46].

The Cd^2+^ absorbed by plants is accumulated in the roots. This accumulation of cadmium underground reduces the damage of heavy metals to the shoots. Compared with the Cd group, Cd-AM treatment significantly reduced cadmium ion concentration in *M. truncatula*. The association of mycorrhizae with plants may reduce the transfer of pollutants to plants by serving as a prohibiting barrier that might be able to react by binding metals to the hyphae of fungi [47,48]. The vesicles, spores, extraradical mycelia, and intraradical mycelia of fungi play a vital role in the chelation of pollutants and the accrual of metals [49]. Plant ROS is often induced under abiotic stresses such as absence of light, metals, drought, and flooding [50]. MDA content can reflect the degree of damage to plants [51]. Our findings demonstrated that cadmium stress significantly increased MDA accumulation in *M. truncatula*. However, when inoculated with AMF, the content of MDA was significantly lower than in the Cd group. Reduced lipid peroxidation in AMF-inoculated *M. truncatula* supports the beneficial role of AMF in plants subjected to cadmium stress [52,53]. CAT is present in peroxisomes and glyoxalase bodies and is used to remove hydrogen peroxide from leaves [50]. ASA-GSH is a vital resistance mechanism for plant survival in adversity and plays an essential role in maintaining biofilm stability and defending against membrane lipid peroxidation. MDHAR and GST are critical enzymes in the ASA-GSH cycle that, can effectively scavenge free radicals [50]. The CAT, MDHAR, and GST activities of *M. truncatula* inoculated with AMF under normal physiological conditions were not statistically different from those of the CK group, indicating that the antioxidant enzyme systems of the plants were not activated in these two conditions. Inoculation with AMF under Cd stress reduced the antioxidant activity of *M. truncatula* compared to the Cd group. AMF may reduce the toxicity of cadmium to plants by, among other mechanisms, reducing the accumulation of cadmium in plants. Inoculation of AMF under Cd stress conditions restored soil pH and EC to the level of the CK group. It also significantly increased the soil’s organic matter and carbon content. This indicates that inoculation with AMF can mitigate the harmful effects of heavy metals on the soil and regulate the soil environment to achieve conditions suitable for plant growth [54].

Metabolomics can reveal the metabolic regulation mechanism underlying the observed changes in plant metabolites [55]. Inoculation with AMF under cadmium stress affected the host root system in terms of amino acid metabolism. The upregulation of amino acids such as, L-histidine, leucine, citrulline, L-tryptophan, L-cysteine, homoserine, and methionine indicates that plants can still maintain a regular nitrogen supply under stress and that the accumulation of amino acids favors heavy metal resistance. Amino acid profile changes may indicate a reprogramming of nitrogen metabolism to modulate carbon and nitrogen status, manage plant growth and development, or stimulate defense upon exposure and stress [56,57]. Histidine has been found to chelate metal ions in cells and xylem sap, and changes in its content have functional significance for metal stress tolerance [58,59]. The accumulation of L-cysteine can promote the formation of phytochelatins and metallothioneins, leading to better metal resistance [60]. Homoserine and ethanolamine, which are closely associated with sulfide production in the metabolic pathway, are upregulated, suggesting that AMF inoculation may increase thiol compound secretion in *M. truncatula* roots to increase heavy metal resistance [61,62]. Furthermore, the amino acids glutamine and leucine have been reported to function as signaling molecules and regulate essential stress-responsive genes [63,64]. Inoculating with AMF under abiotic stress may alleviate the stress by synthesizing organic acids, secondary metabolites, and phytohormones, and via other pathways [60,65]. Significantly upregulated expression of organic acids, such as citric acid, tartaric acid, ascorbic acid, ribonic acid, and hexaric acid, which sequester the soil and bind the toxicity of Cd in the soil, can minimize the uptake of Cd by plants and enhance the uptake of essential nutrients by plants [66,67]. Alpha-tocopherol expression was significantly upregulated in the AM-Cd-treated group. Alpha-tocopherol is the most active form of vitamin E and is synthesized in the plastids of higher plants. The study demonstrated that exogenous phenols led to the induction of phenylpropanoids, which could have effectively scavenged reactive oxygen species [68,69]. This study increased salicylic acid contents after inoculation with AMF under cadmium stress. Salicylic acid acts as a signaling molecule involved in several cellular processes in abiotic stress and can be involved in mitigating the harmful effects of heavy metals through signaling cascades [70]. Ahmad showed that salicylic acid helped plants to prevent increased damage due to Cd toxicity and produce an anti-stress response, and attenuated Cd-induced oxidative damage [71,72,73].

## 4. Materials and Methods

### 4.1. Cultivation and Processing of Test Materials

The experimental soil was collected from top layer (0–20 cm) in a farmland area of Southwest University of Science and Technology, Mianyang Sichuan Province, China P.R. (N31°32′2″, E104°41′41″). The collected soil was screened through a 2 mm sieve and sterilized in the high-capacity autoclave sterilizer (TOMY SX-700, USA) at 121 °C lasting for 20 min.The basic physical and chemical properties of the soil were: 1.32 cmol Kg^−1^ CEC, pH 5.03, 11.04 g Kg^−1^ organic matter, 6.38 g Kg^−1^ organic carbon, 43 mg Kg^−1^ Cr, 42 mg Kg^−1^ Cu, 7.7 mg Kg^−1^ Pb, 35.3 mg Kg^−1^ Mn, 0.17 mg Kg^−1^ Hg, and 0.16 mg Kg^−1^ Cd. This test simulated cadmium-contaminated soil under laboratory conditions using CdCl_2_ 5H_2_O as the test cadmium source. A soil concentration of 20 mg/kg it was achieved by adding the cadmium source, which is stable for three weeks. The tested AMF strain (*Glomus mosseae* BGC HUN01A) was provided by the laboratory of the Southwest University of Science and Technology’s Research Center for Material Resources Utilization and Modification Engineering Technology. The above sterilized mixed soil was used to plant *Medicago sativa* in the greenhouse to grow AMF. After about three months, the underground root part was harvested, cultured the soil matrix, and the mixed soil was used as the inoculum for the test [74]. The tested alfalfa was *Medicago truncatula* Gaertn, an annual angiosperm herb of the Leguminosae. The seeds were from breeding garden of School of Life Science and Engineering, Southwestern University of Science and Technology, China P.R.

The entire experiment took place in a glass greenhouse on the roof of the National University Science and Technology Park of the Southwest University of Science and Technology. There were four treatment groups: control (CK), AMF only (AM), Cd only (Cd), and both AMF and Cd (Cd-AM). There were three replicate pots for each treatment. We sterilized the seeds and pots. Each pot contained 1 kg of soil mix, 50 g of microbial inoculum (the same amount of sterilized inoculum as in the control group), and 2 g of *M. truncatula* seeds. The sucrose-wet sieve decantation method detects approximately 100 spores for 1 g of the agent. The root system was stained by the ink vinegar staining method, placed under a microscope for microscopic examination to observe the AMF colonization rate, and the percentage of AM infestation was calculated [60]. Every two days, they were watered with deionized water. Each treatment group was placed randomly in a glasshouse, under natural light, and the greenhouse temperature was controlled at 25 ± 3 °C. After two months of cultivation, plants were harvested and various indicators were measured.

### 4.2. Measurement of Chlorophyll Fluorescence Parameters

After *M. truncatula* had grown for about 60 days, we randomly selected each group of leaves. The chlorophyll fluorescence imaging and related parameters were measured in whole leaves of *M. truncatula* in vivo using a modulated fluorescence imaging system MINI-IMANGING-PAM (WALZ, Effeltrich, Germany). The leaves were subject to dark treatment for 30 min before measurement. Measurement light frequency was set at 1 Hz, intensity at 0.5 µmol/m^−s^, photochemical intensity at 200 µmol/m^−s^, saturation pulse intensity at 2800 µmol/m^−s^, duration at 2 s, and imaging area at 24 mm × 32 mm. The measured parameters included the actual quantum yield Y (II) of photosystem II, the non-photochemical quenching parameters Y (NPQ) and Y (NO), the photochemical quenching coefficient (qL), and the fluorescence imaging of those parameters of *M. truncatula* leaves [75].

### 4.3. Measurement of Antioxidant Enzyme Activity and MDA Content

The fresh leaves were weighed and washed, cut into approximately 0.1 g pieces, and placced into pre-chilled 1.5 mL centrifuge tubes. In this case, 1 mL of pre-chilled phosphate buffer (pH 7.0) was added immediately, and a magnetic bead that had been sterilized overnight in 75% alcohol was placed in each tube with a low-temperature magnetic stirrer. The crude enzyme solution was the supernatant obtained by centrifugation at 10,000 rpm for the 30 s. The activities of catalase (CAT), glutathione sulfhydryltransferase (GST), monodehydroascorbate reductase (MDHAR), and malondialdehyde (MDA) were determined using kits from Nanjing Jiancheng Institute of Biological Engineering.

### 4.4. Measurement of Cd Content in Samples and Physical and Chemical Properties of Soil

Accurately weighed 0.1 g samples from each part of *M. truncatula* in each group, were put into a polyethylene digestion tank, and 5 mLof concentrated nitric acid and 1 mL of 30% hydrogen peroxide were added. The samples were then put it into the graphite digestion instrument (KDNX-20) at 180 °C for 120 min [76]. When the solution was transparent, deionized water was added at 130 °C to remove acid until there was no misty white smoke in the tank. After cooling, it was filtered with a 2 mm organic filter screen and then diluted with deionized water to 10 mL Next, the graphite furnace atomic absorption spectrometer (EWAI AA-7003, China) was used to measure the cadmium content of the above-ground and underground parts of *M. truncatula*.

We took 10 cm of soil from the ground in the flowerpot with a hole punch, mixed the soil five times at random, and placed it in an oven at 60 °C overnight. We then took 0.5 g of soil and measured the soil pH and EC with a pH meter (pHS25, Shanghai, China) and EC meter (CyberScan CON 510, Singapore) according to the national standard (ASTM D2974-14, West Conshohocken, PA, USA). The content of soil organic matter and organic carbon was measured by the potassium dichromate volumetric method.

### 4.5. Root Metabolite Analysis

Plant roots were excised, rinsed with distilled water, frozen with liquid nitrogen, and stored at −80 °C until GC–MS analysis. A 60 mg root sample was weighed, combined with 40 μL L^−2^-chloro-phenylalanine (0.3 mg·mL^−1^ in methanol) and 360 μL of methanol, and milled for 2 min [77]. Next, 200 μL chloroform and 400 μL distilled H_2_O were added, and the sample was ultrasonically extracted for 30 min. The extract was centrifuged at 4 °C and 13,000 rpm for 10 min. A 300 μL sample of the supernatant was transferred to a glass derivatization vial and taken to dryness. An 80 μL volume of methoxamine hydrochloride pyridine solution (15 mg·mL^−1^) was added, and the vial was incubated with shaking for 90 min. An 80 μL volume of N, O-Bis(trimethylsilyl)trifluoroacetamide (BSTFA; CNW, USA), 20 μL hexane, and 10 μL fatty acid methyl ester were added and reacted for60 min at 70 °C. All samples were uniformly mixed in equal amounts and tested according to the above methods. All procedures were subject to quality control (QC). All treatments consisted of three independent biological replicates.

The metabolomics analysis was performed with the following chromatographic conditions: DB-5MS capillary column (30 m × 0.25 mm × 0.25 μm, Agilent J&W Scientific, Folsom, CA, USA), high-purity helium carrier gas, flow rate 1.0 mL·min^−1^, inlet temperature 260 °C, and injection volume 1 μL. The mass spectrometry conditions were an electron impact ion source (EI), full scan mode (SCAN), and a mass scan range of *m*/*z* 50–500.

### 4.6. Statistical Analysis

Data were presented as the means ± SE of independent measurements. Differences among the groups were tested by one-way analysis of variance (ANOVA) followed by Duncan’s new multiple range test at a significance level of *p* < 0.05 using SPSS 18.0. Origin 2018 software was used for basic drawing and information processing. Multivariate statistical analysis was performed using OeBiotech’s free biological information learning platform (https://cloud.oebiotech.cn/task/, accessed on 13 January 2023).

## 5. Conclusions

Inoculating with AMF under cadmium stress can alleviate the damage caused by heavy metals to plants. After inoculation with AMF, the water content and photosynthetic activity of plants increased significantly the uptake of cadmium and the degree of membrane lipid oxidation by plants were reduced, and the soil environment was improved. In the non-targeted metabolic analysis, AMF under Cd stress may promote plant growth by increasing the expression of amino acids to enhance the synthesis of sulfide, increasing the expression of organic acids to reduce the uptake of Cd and mitigate the toxicity of heavy metal ions, and improving the expression of phenols and phytohormones to reduce the accumulation of reactive oxygen species and the damage of heavy metals to plant cell membranes. Based on results from previous studies by others and on our current results, we believe that AMF can alleviate plant stress and promote plant growth. The findings provide evidence to support the potential use of AMF in the phytoremediation of agricultural soils contaminated with toxic metals.

## Figures and Tables

**Table 1:** Shoot and root length, dry weight (DW), and water content of *M. truncatula* under different treatments.

	Shoot Length (cm)	Shoot DW (g plant^−1^)	Shoot Water Content (%)	Root Length (cm)	Root DW (g plant^−1^)	Root Water Content (%)
CK	52.761 ± 3.631 ^a^	0.041 ± 0.003 ^b^	73.18 ^c^	109.341 ± 8.548 ^b^	0.017 ± 0.006 ^a^	53.21 ^a^
AM	64.263 ± 5.582 ^a^	0.074 ± 0.016 ^a^	76.67 ^b^	172.272 ± 10.016 ^a^	0.020 ± 0.002 ^a^	54.48 ^a^
Cd	56.942 ± 7.514 ^a^	0.019 ± 0.002 ^b^	72.99 ^c^	90.799 ± 6.582 ^b^	0.010 ± 0.004 ^a^	52.31 ^a^
Cd-AM	58.353 ± 5.930 ^a^	0.028 ± 0.006 ^b^	81.17 ^a^	111.648 ± 2.141 ^b^	0.012 ± 0.001 ^a^	63.37 ^a^

Values are expressed as means ± SE (*n* = 3). Values followed by the same letter in the same column are not significantly different (*p* < 0.05).

**Table 2:** Content of Cd^2+^ accumulated in the shoot and root of *M. truncatula*.

	Cd^2+^ Content in Shoots (mg/g FW)	Cd^2+^ Content in Roots (mg/g FW)	Shoot-Root Translation Factors
Cd	0.0321 ± 0.0003 ^a^	0.1396 ± 0.0047 ^a^	0.2297 ± 0.0086 ^b^
Cd-AM	0.0293 ± 0.0006 ^b^	0.0683 ± 0.0004 ^b^	0.4297 ± 0.0117 ^a^

Values are expressed as means ± SE (*n* = 3). Values followed by the same letter in the same column are not significantly different (*p* < 0.05).

## Data Availability

Data available on request from the corresponding author.
